# P-2103. Implementation of an Infectious Disease eConsult Service at an Urban Academic Medical Center

**DOI:** 10.1093/ofid/ofaf695.2267

**Published:** 2026-01-11

**Authors:** Ava Hunt, Elena Martin, Blake Gilberto-Bono, Joseph B Ladines-Lim, Anne Norris, Christina J O’Malley, Bhavana Kunisetty, Sara Clemens

**Affiliations:** University of Pennsylvania, Philadelphia, PA; University of Pennsylvania, Philadelphia, PA; Perelman School of Medicine at the University of Pennsylvania, Philadelphia, Pennsylvania; Penn Medicine, Philadelphia, PA; Penn Presbyterian Medical Center, Philadelphia, Pennsylvania; University of Pennsylvania Health System, Philadelphia, Pennsylvania; University of Pennsylvania, Philadelphia, PA; University of Pennsylvania, Philadelphia, PA

## Abstract

**Background:**

Access to infectious disease (ID) consultation is severely limited with ID specialists present in only 20% of United States counties (Walensky et al, PMID: 32491920). Electronic provider-to-provider consultations (eConsults) offer one option for increasing access, previously demonstrating effective delivery of ID services well-received by referring providers (Hofmann et al, PMID: 38813258; Murthy et al, PMID: 28470015).

**Figure 1:**
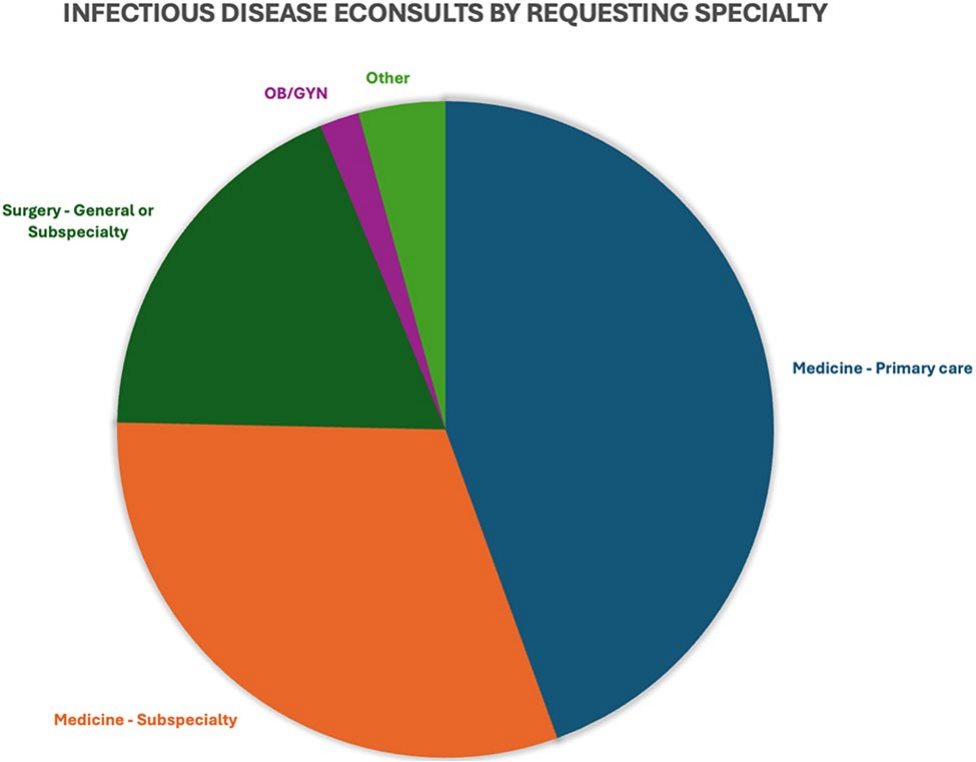
Infectious Disease eConsults by Specialty

**Figure 2:**
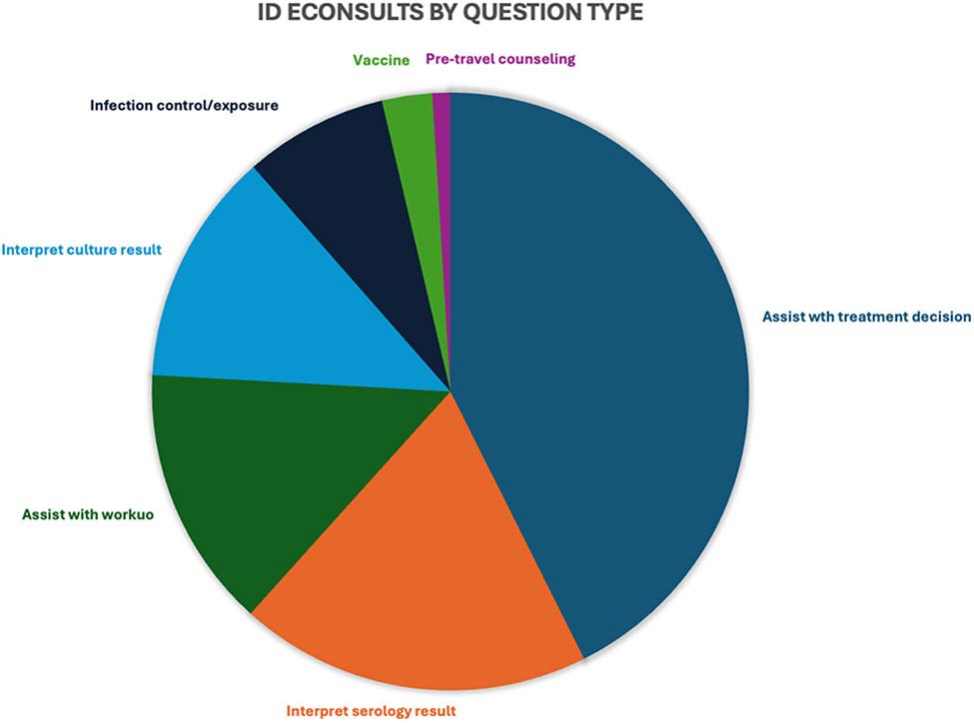
Infectious Disease eConsults by Question Type

**Methods:**

At a large urban academic medical center, an ID eConsult service was offered to providers of all specialties. We analyzed electronic health record data regarding eConsults for quality improvement purposes and distributed a provider satisfaction survey to referring providers.

**Figure 3:**
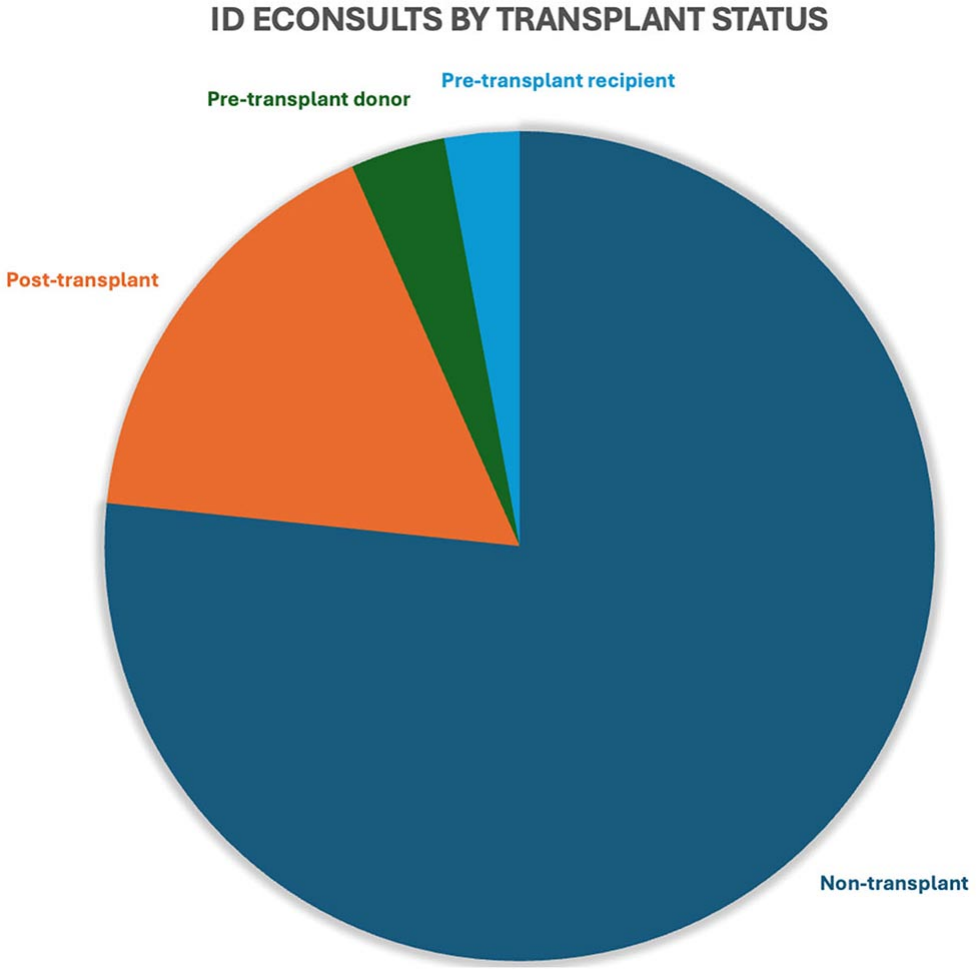
Infectious Disease eConsults by Transplant Status

**Table 1:**
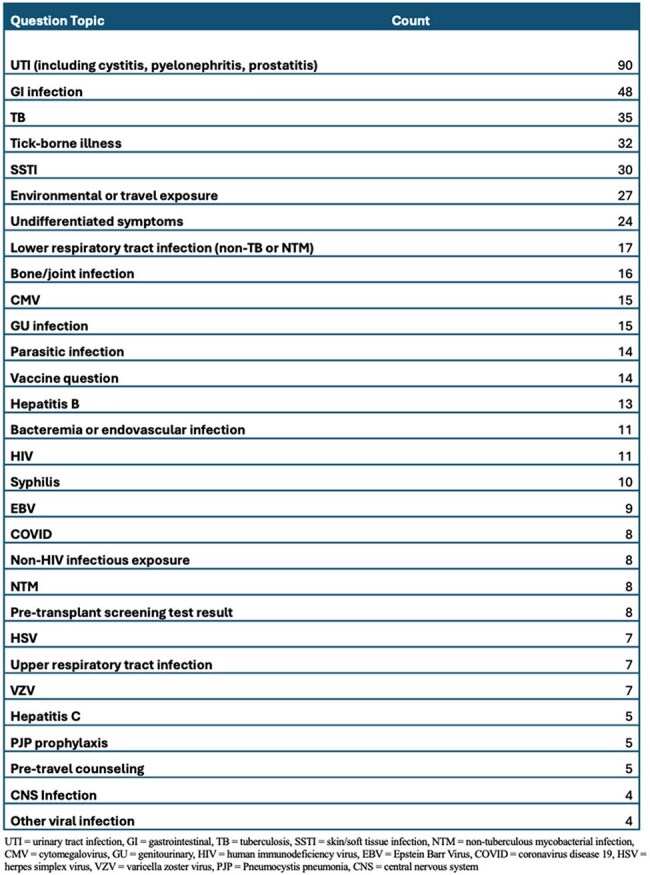
Infectious Disease eConsults by Question Topic

**Results:**

Among 515 ID eConsults over 10 months, 44% were placed by primary care providers, 30% by medical sub-specialists, and 20% by surgeons. Questions on immunocompromised patients comprised 72% of consults, including 24% on solid organ transplant donors or recipients. The most common consult questions concerned therapeutic management (43%), diagnostic management (14%), and interpretation of either culture (13%) or serology (19%) results. The most common topics included urinary tract infection (17%), gastrointestinal infections (9%), tuberculosis (7%), and tick-borne illnesses (6%).

Analysis of reimbursement data showed that 92% of eConsults were billable, covered by 20 of 23 local insurance plans with average reimbursement of $48.82 and average amount of time spent of 7 minutes per eConsult, resulting in a calculated hourly reimbursement rate of $190.66/hr. Most of the 53 survey respondents rated the quality of the service as excellent (89%), were extremely satisfied with the response they received (87%), or would recommend the service to others (89%).

**Conclusion:**

This study is unique in the eConsult literature due to its high proportion of immunocompromised patients including transplant donors and recipients. It also includes a novel financial analysis demonstrating that most eConsults were billable and revenue-generating for the ID division, with a high level of satisfaction by referring providers. Future areas of inquiry include the impact of ID eConsult programs on access to care, consultant/patient satisfaction and antibiotic stewardship.

**Disclosures:**

All Authors: No reported disclosures

